# Plant diseases and pests detection based on deep learning: a review

**DOI:** 10.1186/s13007-021-00722-9

**Published:** 2021-02-24

**Authors:** Jun Liu, Xuewei Wang

**Affiliations:** Shandong Provincial University Laboratory for Protected Horticulture, Blockchain Laboratory of Agricultural Vegetables, Weifang University of Science and Technology, Weifang, 262700 Shandong China

**Keywords:** Deep learning, Convolutional neural network, Plant diseases and pests, Classification, Object detection, Segmentation

## Abstract

Plant diseases and pests are important factors determining the yield and quality of plants. Plant diseases and pests identification can be carried out by means of digital image processing. In recent years, deep learning has made breakthroughs in the field of digital image processing, far superior to traditional methods. How to use deep learning technology to study plant diseases and pests identification has become a research issue of great concern to researchers. This review provides a definition of plant diseases and pests detection problem, puts forward a comparison with traditional plant diseases and pests detection methods. According to the difference of network structure, this study outlines the research on plant diseases and pests detection based on deep learning in recent years from three aspects of classification network, detection network and segmentation network, and the advantages and disadvantages of each method are summarized. Common datasets are introduced, and the performance of existing studies is compared. On this basis, this study discusses possible challenges in practical applications of plant diseases and pests detection based on deep learning. In addition, possible solutions and research ideas are proposed for the challenges, and several suggestions are given. Finally, this study gives the analysis and prospect of the future trend of plant diseases and pests detection based on deep learning.

## Background

Plant diseases and pests detection is a very important research content in the field of machine vision. It is a technology that uses machine vision equipment to acquire images to judge whether there are diseases and pests in the collected plant images [1]. At present, machine vision-based plant diseases and pests detection equipment has been initially applied in agriculture and has replaced the traditional naked eye identification to some extent.

For traditional machine vision-based plant diseases and pests detection method, conventional image processing algorithms or manual design of features plus classifiers are often used [2]. This kind of method usually makes use of the different properties of plant diseases and pests to design the imaging scheme and chooses appropriate light source and shooting angle, which is helpful to obtain images with uniform illumination. Although carefully constructed imaging schemes can greatly reduce the difficulty of classical algorithm design, but also increase the application cost. At the same time, under natural environment, it is often unrealistic to expect the classical algorithms designed to completely eliminate the impact of scene changes on the recognition results [3]. In real complex natural environment, plant diseases and pests detection is faced with many challenges, such as small difference between the lesion area and the background, low contrast, large variations in the scale of the lesion area and various types, and a lot of noise in the lesion image. Also, there are a lot of disturbances when collecting plant diseases and pests images under natural light conditions. At this time, the traditional classical methods often appear helpless, and it is difficult to achieve better detection results.

In recent years, with the successful application of deep learning model represented by convolutional neural network (CNN) in many fields of computer vision (CV, computer-vision), for example, traffic detection [4], medical Image Recognition [5], Scenario text detection [6], expression recognition [7], face Recognition [8], etc. Several plant diseases and pests detection methods based on deep learning are applied in real agricultural practice, and some domestic and foreign companies have developed a variety of deep learning-based plant diseases and pests detection Wechat applet and photo recognition APP software. Therefore, plant diseases and pests detection method based on deep learning not only has important academic research value, but also has a very broad market application prospect.

In view of the lack of comprehensive and detailed discussion on plant diseases and pests detection methods based on deep learning, this study summarizes and combs the relevant literatures from 2014 to 2020, aiming to help researchers quickly and systematically understand the relevant methods and technologies in this field. The content of this study is arranged as follows: “Definition of plant diseases and pests detection problem” section gives the definition of plant diseases and pests detection problem; “Image recognition technology based on deep learning” section focuses on the detailed introduction of image recognition technology based on deep learning; “Plant diseases and pests detection methods based on deep learning” section analyses the three kinds of plant diseases and pests detection methods based on deep learning according to network structure, including classification, detection and segmentation network; “Dataset and performance comparison” section introduces some datasets of plant diseases and pests detection and compares the performance of the existing studies; “Challenges” section puts forward the challenges of plant diseases and pests detection based on deep learning; “Conclusions and future directions” section prospects the possible research focus and development direction in the future.

## Definition of plant diseases and pests detection problem

### Definition of plant diseases and pests

Plant diseases and pests is one kind of natural disasters that affect the normal growth of plants and even cause plant death during the whole growth process of plants from seed development to seedling and to seedling growth. In machine vision tasks, plant diseases and pests tend to be the concepts of human experience rather than a purely mathematical definition.

### Definition of plant diseases and pests detection

**Fig. 1:**
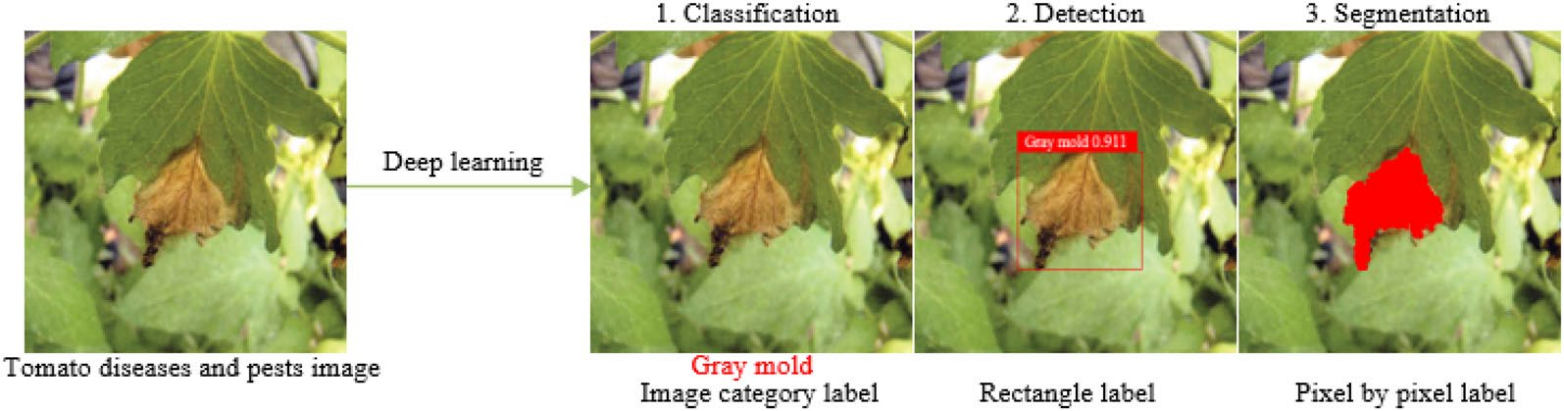
Definition of plant diseases and pests detection problem

Compared with the definite classification, detection and segmentation tasks in computer vision [9], the requirements of plant diseases and pests detection is very general. In fact, its requirements can be divided into three different levels: what, where and how [10]. In the first stage, “what” corresponds to the classification task in computer vision. As shown in Fig. 1, the label of the category to which it belongs is given. The task in this stage can be called classification and only gives the category information of the image. In the second stage, “where” corresponds to the location task in computer vision, and the positioning of this stage is the rigorous sense of detection. This stage not only acquires what types of diseases and pests exist in the image, but also gives their specific locations. As shown in Fig. 1, the plaque area of gray mold is marked with a rectangular box. In the third stage, “how” corresponds to the segmentation task in computer vision. As shown in Fig. 1, the lesions of gray mold are separated from the background pixel by pixel, and a series of information such as the length, area, location of the lesions of gray mold can be further obtained, which can assist the higher-level severity level evaluation of plant diseases and pests. Classification describes the image globally through feature expression, and then determines whether there is a certain kind of object in the image by means of classification operation; while object detection focuses on local description, that is, answering what object exists in what position in an image, so in addition to feature expression, object structure is the most obvious feature that object detection differs from object classification. That is, feature expression is the main research line of object classification, while structure learning is the research focus of object detection. Although the function requirements and objectives of the three stages of plant diseases and pests detection are different, yet in fact, the three stages are mutually inclusive and can be converted. For example, the “where” in the second stage contains the process of “what” in the first stage, and the “how” in the third stage can finish the task of “where” in the second stage. Also, the “what” in the first stage can achieve the goal of the second and the third stages through some methods. Therefore, the problem in this study is collectively referred to as plant diseases and pests detection as conventions in the following text, and the terminology differentiates only when different network structures and functions are adopted.

### Comparison with traditional plant diseases and pests detection methods

To better illustrate the characteristics of plant diseases and pests detection methods based on deep learning, according to existing references [11–15], a comparison with traditional plant diseases and pests detection methods is given from four aspects including essence, method, required conditions and applicable scenarios. Detailed comparison results are shown in Table 1.

**Table 1:** Contrast between traditional image processing methods and deep learning methods

Technology	Traditional image processing methods	Deep learning methods
Essence	Manual design features + classifiers (or rules)	Automatic learning of features from large amounts of data
Method	Image segmentation method: Threshold segmentation; Roberts, Prewitt, Sobel, Laplace and Kirsh edge detection; region segmentation Feature extraction method: SIFT, HOG, LBP, shape, color and texture feature extraction method Classification method: SVM, BP, Bayesian	CNN
Required conditions	Relatively harsh imaging environment requirements, high contrast between lesion and non-lesion areas, less noise	Adequate learning data and high-performance computing units
Applicable scenarios	It is often necessary to change the threshold or redesign the algorithm when imaging environment or plant diseases and pests class changes, which has poor recognition effect in real complex natural environment	It has ability to cope with certain real and complex natural environment changes

## Image recognition technology based on deep learning

Compared with other image recognition methods, the image recognition technology based on deep learning does not need to extract specific features, and only through iterative learning can find appropriate features, which can acquire global and contextual features of images, and has strong robustness and higher recognition accuracy.

### Deep learning theory

The concept of Deep Learning (DL) originated from a paper published in Science by Hinton et al. [16] in 2006. The basic idea of deep learning is: using neural network for data analysis and feature learning, data features are extracted by multiple hidden layers, each hidden layer can be regarded as a perceptron, the perceptron is used to extract low-level features, and then combine low-level features to obtain abstract high-level features, which can significantly alleviate the problem of local minimum. Deep learning overcomes the disadvantage that traditional algorithms rely on artificially designed features and has attracted more and more researchers’ attention. It has now been successfully applied in computer vision, pattern recognition, speech recognition, natural language processing and recommendation systems [17].

Traditional image classification and recognition methods of manual design features can only extract the underlying features, and it is difficult to extract the deep and complex image feature information [18]. And deep learning method can solve this bottleneck. It can directly conduct unsupervised learning from the original image to obtain multi-level image feature information such as low-level features, intermediate features and high-level semantic features. Traditional plant diseases and pests detection algorithms mainly adopt the image recognition method of manual designed features, which is difficult and depends on experience and luck, and cannot automatically learn and extract features from the original image. On the contrary, deep learning can automatically learn features from large data without manual manipulation. The model is composed of multiple layers, which has good autonomous learning ability and feature expression ability, and can automatically extract image features for image classification and recognition. Therefore, deep learning can play a great role in the field of plant diseases and pests image recognition. At present, deep learning methods have developed many well-known deep neural network models, including deep belief network (DBN), deep Boltzmann machine (DBM), stack de-noising autoencoder (SDAE) and deep convolutional neural network (CNN) [19]. In the area of image recognition, the use of these deep neural network models to realize automate feature extraction from high-dimensional feature space offers significant advantages over traditional manual design feature extraction methods. In addition, as the number of training samples grows and the computational power increases, the characterization power of deep neural networks is being further improved. Nowadays, the boom of deep learning is sweeping both industry and academia, and the performance of deep neural network models are all significantly ahead of traditional models. In recent years, the most popular deep learning framework is deep convolutional neural network.

### Convolutional neural network

**Fig. 2:**
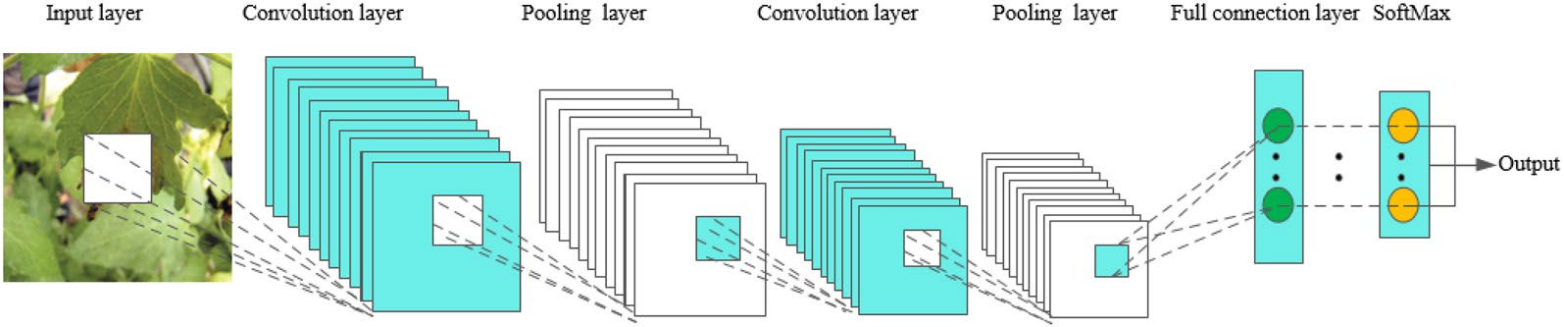
The basic structure of CNN

Convolutional Neural Networks, abbreviated as CNN, has a complex network structure and can perform convolution operations. As shown in Fig. 2, the convolutional neural network model is composed of input layer, convolution layer, pooling layer, full connection layer and output layer. In one model, the convolution layer and the pooling layer alternate several times, and when the neurons of the convolution layer are connected to the neurons of the pooling layer, no full connection is required. CNN is a popular model in the field of deep learning. The reason lies in the huge model capacity and complex information brought about by the basic structural characteristics of CNN, which enables CNN to play an advantage in image recognition. At the same time, the successes of CNN in computer vision tasks have boosted the growing popularity of deep learning.

In the convolution layer, a convolution core is defined first. The convolution core can be considered as a local receptive field, and the local receptive field is the greatest advantage of the convolution neural network. When processing data information, the convolution core slides on the feature map to extract part of the feature information. After the feature extraction of the convolution layer, the neurons are input into the pooling layer to extract the feature again. At present, the commonly used methods of pooling include calculating the mean, maximum and random values of all values in the local receptive field [20, 21]. After the data entering several convolution layers and pooling layers, they enter the full-connection layer, and the neurons in the full-connection layer are fully connected with the neurons in the upper layer. Finally, the data in the full-connection layer can be classified by the softmax method, and then the values are transmitted to the output layer for output results.

### Open source tools for deep learning

The commonly used third-party open source tools for deep learning are Tensorflow [22], Torch/PyTorch [23], Caffe [24], Theano [25]. The different characteristics of each open source tool are shown in Table 2.

**Table 2:** Comparison of open source tools for deep learning

Tools	Publisher	Supporting hardware	Applicable interface	Usability
Tensorflow	Google	CPU, GPU, Mobile	C, Python	Flexible development, portability, powerful performance, support for distributed applications
Torch/PyTorch	Facebook	CPU, GPU, FPGA	C, Python, Lua	Easy to debug and develop, support dynamic neural network, easy to expand, modularization and low learning cost
Caffe	BAIR	CPU, GPU	Python, Matlab	High readability, easy to expand, fast speed, large number of users and wide community
Theano	MILA	CPU, GPU	Python	Flexible and high performance

The four commonly used deep learning third-party open source tools all support cross-platform operation, and the platforms that can be run include Linux, Windows, iOS, Android, etc. Torch/PyTorch and Tensorflow have good scalability and support a large number of third-party libraries and deep network structures, and have the fastest training speed when training large CNN networks on GPU.

## Plant diseases and pests detection methods based on deep learning

**Fig. 3:**
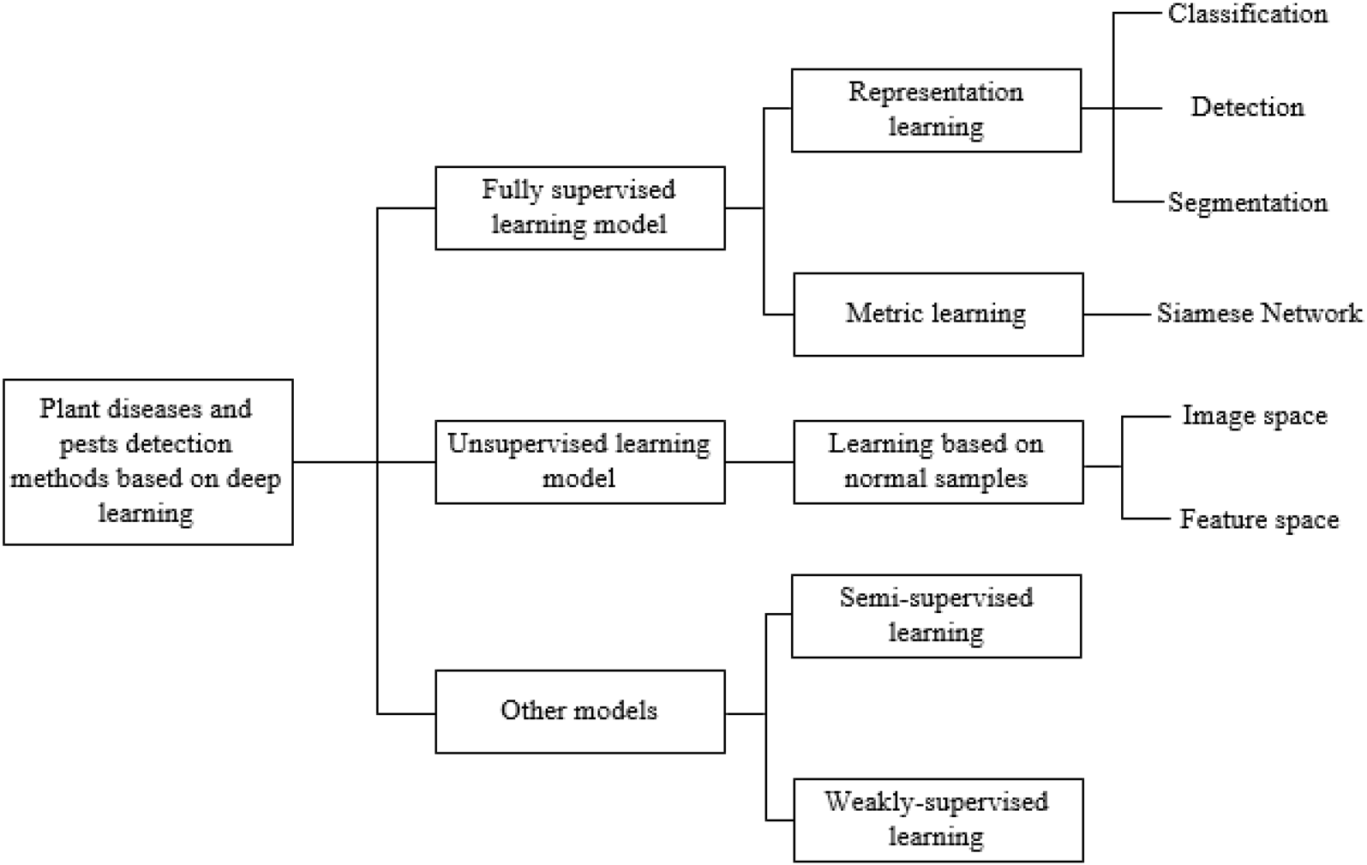
Framework of plant diseases and pests detection methods based on deep learning

This section gives a summary overview of plant diseases and pests detection methods based on deep learning. Since the goal achieved is completely consistent with the computer vision task, plant diseases and pests detection methods based on deep learning can be seen as an application of relevant classical networks in the field of agriculture. As shown in Fig. 3, the network can be further subdivided into classification network, detection network and segmentation network according to the different network structures. As can be seen from Fig. 3, this paper is subdivided into several different sub-methods according to the processing characteristics of each type of methods.

### Classification network

In real natural environment, the great differences in shape, size, texture, color, background, layout and imaging illumination of plant diseases and pests make the recognition a difficult task. Due to the strong feature extraction capability of CNN, the adoption of CNN-based classification network has become the most commonly used pattern in plant diseases and pests classification. Generally, the feature extraction part of CNN classification network consists of cascaded convolution layer + pooling layer, followed by full connection layer (or average pooling layer) + softmax structure for classification. Existing plant diseases and pests classification network mostly use the muture network structures in computer vision, including AlexNet [26], GoogleLeNet [27], VGGNet [28], ResNet [29], Inception V4 [30], DenseNets [31], MobileNet [32] and SqueezeNet [33]. There are also some studies which have designed network structures based on practical problems [34–37]. By inputting a test image into the classification network, the network analyses the input image and returns a label that classifies the image. According to the difference of tasks achieved by the classification network method, it can be subdivided into three subcategories: using the network as a feature extractor, using the network for classification directly and using the network for lesions location.

#### Using network as feature extractor

In the early studies on plant diseases and pests classification methods based on deep learning, many researchers took advantage of the powerful feature extraction capability of CNN, and the methods were combined with traditional classifiers [38]. First, the images are input into a pretrained CNN network to obtain image characterization features, and the acquired features are then input into a conventional machine learning classifier (e.g., SVM) for classification. Yalcin et al. [39] proposed a convolutional neural network architecture to extract the features of images while performing experiments using SVM classifiers with different kernels and feature descriptors such as LBP and GIST, the experimental results confirmed the effectiveness of the approach. Fuentes et al. [40] put forward the idea of CNN based meta architecture with different feature extractors, and the input images included healthy and infected plants, which were identified as their respective classes after going through the meta architecture. Hasan et al. [41] identified and classified nine different types of rice diseases by using the features extracted from DCNN model and input into SVM, and the accuracy achieved 97.5%.

#### Using network for classification directly

Directly using classification network to classify lesions is the earliest common means of CNN applied in plant diseases and pests detection. According to the characteristics of existing research work, it can be further subdivided into original image classification, classification after locating Region of Interest (ROI) and multi-category classification.

1. Original image classification. That is, directly put the collected complete plant diseases and pests image into the network for learning and training. Thenmozhi et al. [42] proposed an effective deep CNN model, and transfer learning is used to fine-tune the pre-training model. Insect species were classified on three public insect datasets with accuracy of 96.75%, 97.47% and 95.97%, respectively. Fang et al. [43] used ResNet50 in plant diseases and pests detection. The focus loss function was used instead of the standard cross-entropy loss function, and the Adam optimization method was used to identify the leaf disease grade, and the accuracy achieved 95.61%.
2. Classification after locating ROI. For the whole image acquired, we should focus on whether there is a lesion in a fixed area, so we often obtain the region of interest (ROI) in advance, and then input the ROI into the network to judge the category of diseases and pests. Nagasubramanian et al. [44] used a new three-dimensional deep convolution neural network (DCNN) and salience map visualization method to identify healthy and infected samples of soybean stem rot, and the classification accuracy achieved 95.73%.
3. Multi-category classification. When the number of plant diseases and pests class to be classified exceed 2 class, the conventional plant diseases and pests classification network is the same as the original image classification method, that is, the output nodes of the network are the number of plant diseases and pests class + 1 (including normal class). However, multi-category classification methods often use a basic network to classify lesions and normal samples, and then share feature extraction parts on the same network to modify or increase the classification branches of lesion categories. This approach is equivalent to preparing a pre-training weight parameter for subsequent multi-objective plant diseases and pests classification network, which is obtained by binary training between normal samples and plant diseases and pests samples. Picon et al. [45] proposed a CNN architecture to identify 17 diseases in 5 crops, which seamlessly integrates context metadata, allowing training of a single multi-crop model. The model can achieve the following goals: (a) obtains richer and more robust shared visual features than the corresponding single crop; (b) is not affected by different diseases in which different crops have similar symptoms; (c) seamlessly integrates context to perform crop conditional disease classification. Experiments show that the proposed model alleviates the problem of data imbalance, and the average balanced accuracy is 0.98, which is superior to other methods and eliminates 71% of classifier errors.

#### Using network for lesions location

Generally, the classification network can only complete the classification of image label level. In fact, it can also achieve the location of lesions and the pixel-by-pixel classification by combining different techniques and methods. According to the different means used, it can be further divided into three forms: sliding window, heatmap and multi-task learning network.

1. Sliding window. This is the simplest and intuitive method to achieve the location of lesion coarsely. The image in the sliding window is input into the classification network for plant diseases and pests detection by redundant sliding on the original image through a smaller size window. Finally, all sliding windows are connected to obtain the results of the location of lesion. Chen et al. [46] used CNN classification network based on sliding window to build a framework for characteristics automatic learning, feature fusion, recognition and location regression calculation of plant diseases and pests species, and the recognition rate of 38 common symptoms in the field was 50–90%.
2. Heatmap. This is an image that reflects the importance of each region in the image, the darker the color represents the more important. In the field of plant diseases and pests detection, the darker the color in the heatmap represents the greater the probability that it is the lesion. In 2017, Dechant et al. [47] trained CNN to make heatmap to show the probability of infection in each region in maize disease images, and these heatmaps were used to classify the complete images, dividing each image into containing or not containing infected leaves. At runtime, it takes about 2 min to generate a heatmap for an image (1.6 GB of memory) and less than one second to classify a set of three heatmaps (800 MB of memory). Experiments show that the accuracy is 96.7% on the test dataset. In 2019, Wiesner-Hanks et al. [48] used heatmap method to obtain accurate contour areas of maize diseases, the model can accurately depict lesions as low as millimeter scale from the images collected by UAVs, with an accuracy rate of 99.79%, which is the best scale of aerial plant disease detection achieved so far.
3. Multi-task learning network. If the pure classified network does not add any other skills, it could only realize the image level classification. Therefore, to accurately locate the location of plant diseases and pests, the designed network should often add an extra branch, and the two branches would share the results of the feature extracting. In this way, the network generally had the classification and segmentation output of the plant diseases and pests, forming a multi-task learning network. It takes into account the characteristics of both network. For segmentation network branches, each pixel in the image can be used as a training sample to train the network. Therefore, the multi-task learning network not only uses the segmentation branches to output the specific segmentation results of the lesions, but also greatly reduces the requirements of the classification network for samples. Ren et al. [49] constructed a Deconvolution-Guided VGNet (DGVGNet) model to identify plant leaf diseases which were easily disturbed by shadows, occlusions and light intensity. The deconvolution was used to guide the CNN classifier to focus on the real lesion sites. The test results show that the accuracy of disease class identification is 99.19%, the pixel accuracy of lesion segmentation is 94.66%, and the model has good robustness in occlusion, low light and other environments.

To sum up, the method based on classification network is widely used in practice, and many scholars have carried out application research on the classification of plant diseases and pests [50–53]. At the same time, different sub-methods have their own advantages and disadvantages, as shown in Table 3.

**Table 3:** Comparison of advantages and disadvantages of each sub-method of classification network

Method	Advantages	Disadvantages
Using network as feature extractor	Obtaining effective lesion features	Relying on other classifiers for final classification results
Original image classification	Classic in structure, it is also the basis of other classification network sub-methods and can refer to many existing networks	Lesions need to account for a certain proportion in the image, otherwise their characteristics are easily pooled out, and generally only one class of lesion is allowed in an image
Classification after locating ROI	Obtaining ROI information of the lesions	Additional methods are needed to obtain ROI
Multi-category classification	Solving sample imbalance to some extent	Secondary training is needed
Sliding window	Get rough localization of lesions in images	Sliding window size requires accurate selection, and can only get rough position, slow speed of traversal and sliding
Heatmap	Generate more accurate lesion areas	Accurate lesions location depends on network classification performance
Multi-task learning network	Combining other networks to obtain exact location and category of lesions simultaneously, and reducing the number of training samples required	The network structure is relatively complex, and a pixel-by-pixel label is required when adding segmentation branches

### Detection network

Object positioning is one of the most basic tasks in the field of computer vision. It is also the closest task to plant diseases and pests detections in the traditional sense. Its purpose is to obtain accurate location and category information of the object. At present, object detection methods based on deep learning emerge endlessly. Generally speaking, plant diseases and pests detection network based on deep learning can be divided into: two stage network represented by Faster R-CNN [54]; one stage network represented by SSD [55] and YOLO [56–58]. The main difference between the two networks is that the two-stage network needs to first generate a candidate box (proposal) that may contain the lesions, and then further execute the object detection process. In contrast, the one-stage network directly uses the features extracted in the network to predict the location and class of the lesions.

#### Plant diseases and pests detection based on two stages network

The basic process of two-stage detection network (Faster R-CNN) is to obtain the feature map of the input image through the backbone network first, then calculate the anchor box confidence using RPN and get the proposal. Then, input the feature map of the proposal area after ROIpooling to the network, fine-tune the initial detection results, and finally get the location and classification results of the lesions. Therefore, according to the characteristics of plant diseases and pests detection, common methods often improve on the backbone structure or its feature map, anchor ratio, ROIpooling and loss function. In 2017, Fuentes et al. [59] first used Faster R-CNN to locate tomato diseases and pests directly, combined with deep feature extractors such as VGG-Net and ResNet, the mAP value reached 85.98% in a dataset containing 5000 tomato diseases and pests of 9 categories. In 2019, Ozguven et al. [60] proposed a Faster R-CNN structure for automatic detection of beet leaf spot disease by changing the parameters of CNN model. 155 images were trained and tested. The results show that the overall correct classification rate of this method is 95.48%. Zhou et al. [61] presented a fast rice disease detection method based on the fusion of FCM-KM and Faster R-CNN. The application results of 3010 images showed that: the detection accuracy and time of rice blast, bacterial blight, and sheath blight are 96.71%/0.65 s, 97.53%/0.82 s and 98.26%/0.53 s respectively. Xie et al. [62] proposed a Faster DR-IACNN model based on the self-built grape leaf disease dataset (GLDD) and Faster R-CNN detection algorithm, the Inception-v1 module, Inception-ResNet-v2 module and SE are introduced. The proposed model achieved higher feature extraction ability, the mAP accuracy was 81.1% and the detection speed was 15.01FPS. The two-stage detection network has been devoted to improving the detection speed to improve the real-time and practicability of the detection system, but compared with the single-stage detection network, it is still not concise enough, and the inference speed is still not fast enough.

#### Plant diseases and pests detection based on one stage network

The one-stage object detection algorithm has eliminated the region proposal stage, but directly adds the detection head to the backbone network for classification and regression, thus greatly improving the inference speed of the detection network. The single-stage detection network is divided into two types, SSD and YOLO, both of which use the whole image as the input of the network, and directly return the position of the bounding box and the category to which it belongs at the output layer.

Compared with the traditional convolutional neural network, the SSD selects VGG16 as the trunk of the network, and adds a feature pyramid network to obtain features from different layers and make predictions. Singh et al. [63] built the PlantDoc dataset for plant disease detection. Considering that the application should predict in mobile CPU in real time, an application based on MobileNets and SSD was established to simplify the detection of model parameters. Sun et al. [64] presented an instance detection method of multi-scale feature fusion based on convolutional neural network, which is improved on the basis of SSD to detect maize leaf blight under complex background. The proposed method combined data preprocessing, feature fusion, feature sharing, disease detection and other steps. The mAP of the new model is higher (from 71.80 to 91.83%) than that of the original SSD model. The FPS of the new model has also improved (from 24 to 28.4), reaching the standard of real-time detection.

YOLO considers the detection task as a regression problem, and uses global information to directly predict the bounding box and category of the object to achieve end-to-end detection of a single CNN network. YOLO can achieve global optimization and greatly improve the detection speed while satisfying higher accuracy. Prakruti et al. [65] presented a method to detect pests and diseases on images captured under uncontrolled conditions in tea gardens. YOLOv3 was used to detect pests and diseases. While ensuring real-time availability of the system, about 86% mAP was achieved with 50% IOU. Zhang et al. [66] combined the pooling of spatial pyramids with the improved YOLOv3, deconvolution is implemented by using the combination of up-sampling and convolution operation, which enables the algorithm to effectively detect small size crop pest samples in the image and reduces the problem of relatively low recognition accuracy due to the diversity of crop pest attitudes and scales. The average recognition accuracy can reach 88.07% by testing 20 class of pests collected in real scene.

In addition, there are many studies on using detection network to identify diseases and pests [47, 67–73]. With the development of object detection network in computer vision, it is believed that more and more new detection models will be applied in plant diseases and pests detection in the future. In summary, in the field of plant diseases and pests detection which emphasizes detection accuracy at this stage, more models based on two-stage are used, and in the field of plant diseases and pests detection which pursue detection speed more models based on one-stage are used.

Can detection network replace classification network? The task of detection network is to solve the location problem of plant diseases and pests. The task of classification network is to judge the class of plant diseases and pests. Visually, the hidden information of detection network includes the category information, that is, the category information of plant diseases and pests that need to be located needs to be known beforehand, and the corresponding annotation information should be given in advance to judge the location of plant diseases and pests. From this point of view, the detection network seems to include the steps of the classification network, that is, the detection network can answer “what kind of plant diseases and pests are in what place”. But there is a misconception, in which “what kind of plant diseases and pests” is given a priori, that is, what is labelled during training is not necessarily the real result. In the case of strong model differentiation, that is, when the detection network can give accurate results, the detection network can answer “what kind of plant diseases and pests are in what place” to a certain extent. However, in the real world, in many cases, it cannot uniquely reflect the uniqueness of plant diseases and pests categories, only can answer “what kind of plant diseases and pests may be in what place”, then the involvement of the classification network is necessary. Thus, the detection network cannot replace the classification network.

### Segmentation network

Segmentation network converts the plant diseases and pests detection task to semantic and even instance segmentation of lesions and normal areas. It not only finely divides the lesion area, but also obtains the location, category and corresponding geometric properties (including length, width, area, outline, center, etc.). It can be roughly divided into: Fully Convolutional Networks (FCN) [74] and Mask R-CNN [75].

#### FCN

Full convolution neural network (FCN) is the basis of image semantics segmentation. At present, almost all semantics segmentation models are based on FCN. FCN first extracts and codes the features of the input image using convolution, then gradually restores the feature image to the size of the input image by deconvolution or up sampling. Based on the differences in FCN network structure, the plant diseases and pests segmentation methods can be divided into conventional FCN, U-net [76] and SegNet [77].

1. Conventional FCN. Wang et al. [78] presented a new method of maize leaf disease segmentation based on full convolution neural network to solve the problem that traditional computer vision is susceptible to different illumination and complex background, and the segmentation accuracy reached 96.26. Wang et al. [79] proposed a plant diseases and pests segmentation method based on improved FCN. In this method, a convolution layer was used to extract multi-layer feature information from the input maize leaf lesion image, and the size and resolution of the input image were restored by deconvolution operation. Compared with the original FCN method, not only the integrity of the lesion was guaranteed, but also the segmentation of small lesion area was highlighted, and the accuracy rate reached 95.87%.
2. U-net. U-net is not only a classical FCN structure, but also a typical encoder-decoder structure. It is characterized by introducing a layer-hopping connection, fusing the feature map in the coding stage with that in the decoding stage, which is beneficial to the recovery of segmentation details. Lin et al. [80] used U-net based convolutional neural network to segment 50 cucumber powdery mildew leaves collected in natural environment. Compared with the original U-net, a batch normalization layer was added behind each convolution layer, making the neural network insensitive to weight initialization. The experiment shows that the convolutional neural network based on U-net can accurately segment powdery mildew on cucumber leaves at the pixel level with an average pixel accuracy of 96.08%, which is superior to the existing K-means, Random-forest and GBDT methods. The U-net method can segment the lesion area in a complex background, and still has good segmentation accuracy and segmentation speed with fewer samples.
3. SegNet. It is also a classical encoder–decoder structure. Its feature is that the up-sampling operation in the decoder takes advantage of the index of the largest pooling operation in the encoder. Kerkech et al. [81] presented an image segmentation method for unmanned aerial vehicles. Visible and infrared images (480 samples from each range) were segmented using SegNet to identify four categories: shadows, ground, healthy and symptomatic grape vines. The detection rates of the proposed method on grape vines and leaves were 92% and 87%, respectively.

#### Mask R-CNN

Mask R-CNN is one of the most commonly used image instance segmentation methods at present. It can be considered as a multitask learning method based on detection and segmentation network. When multiple lesions of the same type have adhesion or overlap, instance segmentation can separate individual lesions and further count the number of lesions. However, semantic segmentation often treats multiple lesions of the same type as a whole. Stewart et al. [82] trained a Mask R-CNN model to segment maize northern leaf blight (NLB) lesions in an unmanned aerial vehicle image. The trained model can accurately detect and segment a single lesion. At the IOU threshold of 0.50, the IOU between the baseline true value and the predicted lesion was 0.73, and the average accuracy was 0.96. Also, some studies combine the Mask R-CNN framework with object detection networks for plant diseases and pests detection. Wang et al. [83] used two different models, Faster R-CNN and ask R-CNN, in which Faster R-CNN was used to identify the class of tomato diseases and Mask R-CNN was used to detect and segment the location and shape of the infected area. The results showed that the proposed model can quickly and accurately identify 11 class of tomato diseases, and divide the location and shape of infected areas. Mask R-CNN reached a high detection rate of 99.64% for all class of tomato diseases.

Compared with the classification and detection network methods, the segmentation method has advantages in obtaining the lesion information. However, like the detection network, it requires a lot of annotation data, and its annotation information is pixel by pixel, which often takes a lot of effort and cost.

## Dataset and performance comparison

This section first gives a brief introduction to the plant diseases and pests related datasets and the evaluation index of deep learning model, then compares and analyses the related models of plant diseases and pests detection based on deep learning in recent years.

### Datasets for plant diseases and pests detection

Plant diseases and pests detection datasets are the basis for research work. Compared with ImageNet, PASCAL-VOC2007/2012 and COCO in computer vision tasks, there is not a large and unified dataset for plant diseases and pests detection. The plant diseases and pests dataset can be acquired by self-collection, network collection and use of public datasets. Among them, self-collection of image dataset is often obtained by unmanned aerial remote sensing, ground camera photography, Internet of Things monitoring video or video recording, aerial photography of unmanned aerial vehicle with camera, hyperspectral imager, near-infrared spectrometer, and so on. Public datasets typically come from PlantVillage, an existing well-known public standard library. Relatively, self-collected datasets of plant diseases and pests in real natural environment are more practical. Although more and more researchers have opened up the images collected in the field, it is difficult to compare them uniformly based on different class of diseases under different detection objects and scenarios. This section provides links to a variety of plant diseases and pests detection datasets in conjunction with existing studies. As shown in Table 4.

**Table 4:** Common datasets for plant diseases and pests detection

Species	Collection environment	Link
PlantVillage-Dataset: 50,000 images of classified plant diseases of 14 crop varieties and 26 diseases [84]	Detached leaves on a plain background	https://github.com/spMohanty/PlantVillage-Dataset
Rice Leaf Diseases Data Set: three classes of diseases: Bacterial leaf blight, Brown spot, and Leaf smut, each having 40 images [85, 86]	Captured with a white background in direct sunlight	https://archive.ics.uci.edu/ml/datasets/Rice+Leaf+Diseases
Image Database for Plant Disease Symptoms (PDDB): 2326 images of 171 diseases and other disorders affecting 21 plant species [87]	Field	https://www.digipathos-rep.cnptia.embrapa.br
New Plant Diseases Dataset (Augmented): 87 K rgb images of healthy and diseased crop leaves which is categorized into 38 different classes	Detached leaves on a plain background	https://www.kaggle.com/vipoooool/new-plant-diseases-dataset/
38 disease classes from PlantVillage dataset and 1 background class from Stanford’s open dataset of background images—DAGS [88]	Network	https://github.com/MarkoArsenovic/DeepLearning_PlantDiseases
18,222 images annotated with 105,705 northern leaf blight (NLB) lesions [89]	Field	https://osf.io/p67rz/
40 classes of insects from rice, maize, soybean, sugarcane and cotton crops	Field	http://www.nbair.res.in/insectpests/pestsearch.php
17,624 high quality JPG image data of rice, wheat and maize of 200 GB	Field	http://www.icgroupcas.cn/website_bchtk/index.html
PlantDoc dataset: 2598 data points in total across 13 plant species and up to 17 classes of diseases [63]	Field	https://github.com/pratikkayal/PlantDoc-Object-Detection-Dataset https://github.com/pratikkayal/PlantDoc-Dataset
Northern Leaf Blight (NLB) dataset for Maize	Field	https://bisque.cyverse.org/client_service/browser?resource=/data_service/dataset
3651 images of apple leaf disease [90]	Field	https://www.kaggle.com/c/plantpathology-2020-fgvc7
IP102: Insect Pest Recognition Database: 75,000 images belonging to 102 categories [91]	Field	https://github.com/xpwu95/IP102
A database of eight common tomato pest images [92]	Network	https://data.mendeley.com/datasets/s62zm6djd2/1

### Evaluation indices

Evaluation indices can vary depending on the focus of the study. Common evaluation indices include $Precision$, $Recall$, mean Average Precision (mAP) and the harmonic Mean F1 score based on $Precision$ and $Recall$.

$Precision$ and $Recall$ are defined as:

1 $$Precision = \frac{TP}{{TP + FP}} \cdot 100\% ,$$

2 $$Recall = \frac{TP}{{TP + FN}} \cdot 100\% .$$

In Formula (1) and Formula (2), TP (True Positive) is true-positive, predicted to be 1 and actually 1, indicating the number of lesions correctly identified by the algorithm. FP (False Positive) is false-positive, predicted to be 1 and actually 0, indicating the number of lesions incorrectly identified by the algorithm. FN (False Negative) is false-negative, predicted to be 0 and actually 1, indicating the number of unrecognized lesions.

Detection accuracy is usually assessed using mAP. The average accuracy of each category in the dataset needs to be calculated first:

3 $$P_{average} = \mathop \sum \limits_{j = 1}^{{N\left( {class} \right)}} Precision\left( j \right) \cdot Recall\left( j \right) \cdot 100\% .$$

In the above-mentioned formula, $N\left( {class} \right)$ represents the number of all categories, $Precision\left( j \right)$ and $Recall\left( j \right)$ represents the precision and recall of class *j* respectively.

Average accuracy for each category is defined as mAP:

4 $$mAP = \frac{{P_{average} }}{{N\left( {class} \right)}}.$$

The greater the value of $mAP$, the higher the recognition accuracy of the algorithm; conversely, the lower the accuracy of the algorithm.

F1 score is also introduced to measure the accuracy of the model. F1 score takes into account both the accuracy and recall of the model. The formula is

5 $${\text{F1}} = \frac{2 Precision \cdot Recall}{{Precision + Recall}} \cdot 100\% .$$

Frames per second (FPS) is used to evaluate the recognition speed. The more frames per second, the faster the algorithm recognition speed; conversely, the slower the algorithm recognition speed.

### Performance comparison of existing algorithms

At present, the research on plant diseases and pests based on deep learning involves a wide range of crops, including all kinds of vegetables, fruits and food crops. The tasks completed include not only the basic tasks of classification, detection and segmentation, but also more complex tasks such as the judgment of infection degree.

At present, most of the current deep learning-based methods for plant diseases and pests detection are applied on specific datasets, many datasets are not publicly available, there is still no single publicly available and comprehensive dataset that will allow all algorithms to be uniformly compared. With the continuous development of deep learning, the application performance of some typical algorithms on different datasets has been gradually improved, and the mAP, F1 score and FPS of the algorithms have all been increased.

The breakthroughs achieved in the existing studies are amazing, but due to the fact that there is still a certain gap between the complexity of the infectious diseases and pests images in the existing studies and the real-time field diseases and pests detection based on mobile devices. Subsequent studies will need to find breakthroughs in larger, more complex, and more realistic datasets.

## Challenges

### Small dataset size problem

At present, deep learning methods are widely used in various computer vision tasks, plant diseases and pests detection is generally regarded as specific application in the field of agriculture. There are too few agricultural plant diseases and pests samples available. Compared with open standard libraries, self-collected data sets are small in size and laborious in labeling data. Compared with more than 14 million sample data in ImageNet datasets, the most critical problem facing plant diseases and pests detection is the problem of small samples. In practice, some plant diseases have low incidence and high cost of disease image acquisition, resulting in only a few or dozen training data collected, which limits the application of deep learning methods in the field of plant diseases and pests identification. In fact, for the problem of small samples, there are currently three different solutions.

#### Data amplification, synthesis and generation

Data amplification is a key component of training deep learning models. An optimized data amplification strategy can effectively improve the plant diseases and pests detection effect. The most common method of plant diseases and pests image expansion is to acquire more samples using image processing operations such as mirroring, rotating, shifting, warping, filtering, contrast adjustment, and so on for the original plant diseases and pests samples. In addition, Generative Adversarial Networks (GANs) [93] and Variational automatic encoder (VAE) [94] can generate more diverse samples to enrich limited datasets.

#### Transfer learning and fine-tuning classical network model

Transfer learning (TL) transfers knowledge learned from generic large datasets to specialized areas with relatively small amounts of data. When transfer learning develops a model for newly collected unlabeled samples, it can start with a training model by a similar known dataset. After fine-tuning parameters or modifying components, it can be applied to localized plant disease and pest detection, which can reduce the cost of model training and enable the convolution neural network to adapt to small sample data. Oppenheim et al. [95] collected infected potato images of different sizes, hues and shapes under natural light and classified by fine-tuning the VGG network. The results showed that, the transfer learning and training of new networks were effective. Too et al. [96] evaluated various classical networks by fine-tuning and contrast. The experimental results showed that the accuracy of Dense-Nets improved with the number of iterations. Chen et al. [97] used transfer learning and fine-tuning to identify rice disease images under complex background conditions and achieved an average accuracy of 92.00%, which proves that the performance of transfer learning is better than training from scratch.

#### Reasonable network structure design

By designing a reasonable network structure, the sample requirements can be greatly reduced. Zhang et al. [98] constructed a three-channel convolution neural network model for plant leaf disease recognition by combining three color components. Each channel TCCNN component is composed of three color RGB leaf disease images. Liu et al. [99] presented an improved CNN method for identifying grape leaf diseases. The model used a depth-separable convolution instead of a standard convolution to alleviate overfitting and reduce the number of parameters. For the different size of grape leaf lesions, the initial structure was applied to the model to improve the ability of multi-scale feature extraction. Compared with the standard ResNet and GoogLeNet structures, this model has faster convergence speed and higher accuracy during training. The recognition accuracy of this algorithm was 97.22%.

### Fine-grained identification of small-size lesions in early identification

#### Small-size lesions in early identification

Accurate early detection of plant diseases is essential to maximize the yield [36]. In the actual early identification of plant diseases and pests, due to the small size of the lesion object itself, multiple down sampling processes in the deep feature extraction network tend to cause small-scale objects to be ignored. Moreover, due to the background noise problem on the collected images, large-scale complex background may lead to more false detection, especially on low-resolution images. In view of the shortage of existing algorithms, the improvement direction of small object detection algorithm is analyzed, and several strategies such as attention mechanism are proposed to improve the performance of small target detection.

The use of attention mechanism makes resources allocated more rationally. The essence of attention mechanism is to quickly find region of interest and ignore unimportant information. By learning the characteristics of plant diseases and pests images, features can be separated using weighted sum method with weighted coefficient, and the background noise in the image can be suppressed. Specifically, the attention mechanism module can get a salient image, and seclude the object from the background, and the Softmax function can be used to manipulate the feature image, and combine it with the original feature image to obtain new fusion features for noise reduction purposes. In future studies on early recognition of plant diseases and pests, attention mechanisms can be used to effectively select information and allocate more resources to region of interest to achieve more accurate detection. Karthik et al. [100] applied attention mechanism on the residual network and experiments were carried out using the plantVillage dataset, which achieved 98% overall accuracy.

#### Fine-grained identification

First, there is a large difference within the class, that is, the visual characteristics of plant diseases and pests belonging to the same class are quite different. The reason is that the aforementioned external factors such as uneven illumination, dense occlusion, blurred equipment dithering and other interferences, resulting in different image samples belonging to the same kind of diseases and pests differ greatly. Plant diseases and pests detection in complex scenarios is a very challenging task of fine-grained recognition [101]. The existence of growth variations of diseases and pests results in distinct differences in the characterization of the same diseases and pests at different stages, forming the “intra-class difference” fine-grained characteristics.

Secondly, there is fuzziness between classes, that is, objects of different classes have some similarity. There are many detailed classifications of biological subspecies and subclasses of different kinds of diseases and pests, and there are some similarities of biological morphology and life habits among the subclasses, which lead to the problem of fine-grained identification of “inter-class similarity”. Barbedo believed that similar symptoms could be produced, which even phytopathologists could not correctly distinguish [102].

Thirdly, background disturbance makes it impossible for plant diseases and pests to appear in a very clean background in the real world. Background can be very complex and interfere with objects of interest, which makes plant diseases and pests detection more difficult. Some literature often ignores this issue because images are captured under controlled conditions [103].

Relying on the existing deep learning methods can not effectively identify the fine-grained characteristics of diseases and pests that exist naturally in the application of the above actual agricultural scenarios, resulting in technical difficulties such as low identification accuracy and generalization robustness, which has long restricted the performance improvement of decision-making management of diseases and pests by the Intelligent Agricultural Internet of Things [104]. The existing research is only suitable for fine-grained identification of fewer class of diseases and pests, can not solve the problem of large-scale, large-category, accurate and efficient identification of diseases and pests, and is difficult to deploy directly to the mobile terminals of smart agriculture.

### Detection performance under the influence of illumination and occlusion

#### Lighting problems

Previous studies have collected images of plant diseases and pests mostly in indoor light boxes [105]. Although this method can effectively eliminate the influence of external light to simplify image processing, it is quite different from the images collected under real natural light. Because natural light changes very dynamically, and the range in which the camera can accept dynamic light sources is limited, it is easy to cause image color distortion when above or below this limit. In addition, due to the difference of view angle and distance during image collection, the apparent characteristics of plant diseases and pests change greatly, which brings great difficulties to the visual recognition algorithm.

#### Occlusion problem

At present, most researchers intentionally avoid the recognition of plant diseases and pests in complex environments. They only focus on a single background. They use the method of directly intercepting the area of interest to the collected images, but seldom consider the occlusion problem. As a result, the recognition accuracy under occlusion is low and the practicability is greatly reduced. Occlusion problems are common in real natural environments, including blade occlusion caused by changes in blade posture, branch occlusion, light occlusion caused by external lighting, and mixed occlusion caused by different types of occlusion. The difficulties of plant diseases and pests identification under occlusion are the lack of features and noise overlap caused by occlusion. Different occlusion conditions have different degrees of impact on the recognition algorithm, resulting in false detection or even missed detection. In recent years, with the maturity of deep learning algorithms under restricted conditions, some researchers have gradually challenged the identification of plant diseases and pests under occluded conditions [106, 107], and significant progress has been made, which lays a good foundation for the application of plant diseases and pests identification in real-world scenarios. However, occlusion is random and complex. The training of the basic framework is difficult and the dependence on the performance of hardware devices still exists, we should strengthen the innovation and optimization of the basic framework, including the design of lightweight network architecture. The exploration of GAN and other aspects should be enhanced, while ensuring the accuracy of detection, the difficulty of model training should be reduced. GAN has prominent advantages in dealing with posture changes and chaotic background, but its design is not yet mature, and it is easy to crash in learning and cause model uncontrollable problems during training. We should strengthen the exploration of network performance to make it easier to quantify the quality of the model.

#### Detection speed problem

Compared with traditional methods, deep learning algorithms have better results, but their computational complexity is also higher. If the detection accuracy is guaranteed, the model needs to fully learn the characteristics of the image and increase the computational load, which will inevitably lead to slow detection speed and can not meet the needs of real-time. In order to ensure the detection speed, it is usually necessary to reduce the amount of calculation. However, this will cause insufficient training and result in false or missed detection. Therefore, it is important to design an efficient algorithm with both detection accuracy and detection speed.

Plant diseases and pests detection methods based on deep learning include three main links in agricultural applications: data labeling, model training and model inference. In real-time agricultural applications, more attention is paid to model inference. Currently, most plant diseases and pests detection methods focus on the accuracy of recognition. Little attention is paid to the efficiency of model inference. In reference [108], to improve the efficiency of the model calculation process to meet the actual agricultural needs, a deep separable convolution structure model for plant leaf disease detection was introduced. Several models were trained and tested. The classification accuracy of Reduced MobileNet was 98.34%, the parameters were 29 times less than VGG, and 6 times less than MobileNet. This shows an effective compromise between delay and accuracy, which is suitable for real-time crop diseases diagnosis on resource-constrained mobile devices.

## Conclusions and future directions

Compared with traditional image processing methods, which deal with plant diseases and pests detection tasks in several steps and links, plant diseases and pests detection methods based on deep learning unify them into end-to-end feature extraction, which has a broad development prospects and great potential. Although plant diseases and pests detection technology is developing rapidly, it has been moving from academic research to agricultural application, there is still a certain distance from the mature application in the real natural environment, and there are still some problems to be solved.

### Plant diseases and pests detection dataset

Deep learning technology has made some achievements in the identification of plant diseases and pests. Various image recognition algorithms have also been further developed and extended, which provides a theoretical basis for the identification of specific diseases and pests. However, the collection of image samples in previous studies mostly come from the characterization of disease spots, insect appearance characteristics or the characterization of insect pests and leaves. Most of the research results are limited to the laboratory environment and are applicable only to the plant diseases and pests images obtained at the time. The main reason for this is that the growth of plants is cyclical, continuous, seasonal and regional. Similarly, the characteristics of the same disease or pest at different growing stages of crops are different. Images of different plant species vary from region to region. As a result, most of the existing research results are not universal. Even with a high recognition rate in a single trial, the validity of the data obtained at other times cannot be guaranteed.

Most of the existing studies are based on the images generated in the visible range, but the electromagnetic wave outside the visible range also contains a lot of information, so the comprehensive information such as visible light, near infrared, multi-spectral should be fused to achieve the acquisition of plant diseases and pests dataset. Future research should focus on multi-information fusion method to obtain and identify plant diseases and pests information.

In addition, image databases of different kinds of plant diseases and pests in real natural environments are still in the blank stage. Future research should make full use of the data information acquisition platform such as portable field spore auto-capture instrument, unmanned aerial vehicle aerial photography system, agricultural internet of things monitoring equipment, which performs large-area and coverage identification of farmland and makes up for the lack of randomness of image samples in previous studies. Also, it can ensures the comprehensiveness and accuracy of dataset, and improves the generality of the algorithm.

### Early recognition of plant diseases and pests

In the application of plant diseases and pests identification, the manifestation symptoms are not obvious, so early diagnosis is very difficult whether it is by visual observation or computer interpretation. However, the research significance and demand of early diagnosis are greater, which is more conducive to the prevention and control of plant diseases and pests and prevent their spread and development. The best image quality can be obtained when the sunlight is sufficient, and taking pictures in cloudy weather will increase the complexity of image preprocessing and reduce the recognition effect. In addition, in the early stage of plant diseases and pests occurrence, even high-resolution images are difficult to analyze. It is necessary to combine meteorological and plant protection data such as temperature and humidity to realize the recognition and prediction of diseases and pests. By consulting the existing research literatures, there are few reports on the early diagnosis of plant diseases and pests.

### Network training and learning

When plant diseases and pests are visually identified manually, it is difficult to collect samples of all plant diseases and pests types, and many times only healthy data (positive samples) are available. However, most of the current plant diseases and pests detection methods based on deep learning are supervised learning based on a large number of diseases and pests samples, so manual collection of labelled datasets requires a lot of manpower, so unsupervised learning needs to be explored. Deep learning is a black box, which requires a large number of labelled training samples for end-to-end learning and has poor interpretability. Therefore, how to use the prior knowledge of brain-inspired computing and human-like visual cognitive model to guide the training and learning of the network is also a direction worthy of studying. At the same time, deep models need a large amount of memory and are extremely time-consuming during testing, which makes them unsuitable for deployment on mobile platforms with limited resources. It is important to study how to reduce complexity and obtain fast-executing models without losing accuracy. Finally, the selection of appropriate hyper-parameters has always been a major obstacle to the application of deep learning model to new tasks, such as learning rate, filter size, step size and number, these hyper-parameters have a strong internal dependence, any small adjustment may have a greater impact on the final training results.

### Interdisciplinary research

Only by more closely integrating empirical data with theories such as agronomic plant protection, can we establish a field diagnosis model that is more in line with the rules of crop growth, and will further improve the effectiveness and accuracy of plant diseases and pests identification. In the future, it is necessary to go from image analysis at the surface level to identification of the occurrence mechanism of diseases and pests, and transition from simple experimental environment to practical application research that comprehensively considers crop growth law, environmental factors, etc.

In summary, with the development of artificial intelligence technology, the research focus of plant diseases and pests detection based on machine vision has shifted from classical image processing and machine learning methods to deep learning methods, which solved the difficult problems that could not be solved by traditional methods. There is still a long distance from the popularization of practical production and application, but this technology has great development potential and application value. To fully explore the potential of this technology, the joint efforts of experts from relevant disciplines are needed to effectively integrate the experience knowledge of agriculture and plant protection with deep learning algorithms and models, so as to make plant diseases and pests detection based on deep learning mature. Also, the research results should be integrated into agricultural machinery equipment to truly land the corresponding theoretical results.

## Data Availability

For relevant data and codes, please contact the corresponding author of this manuscript.
